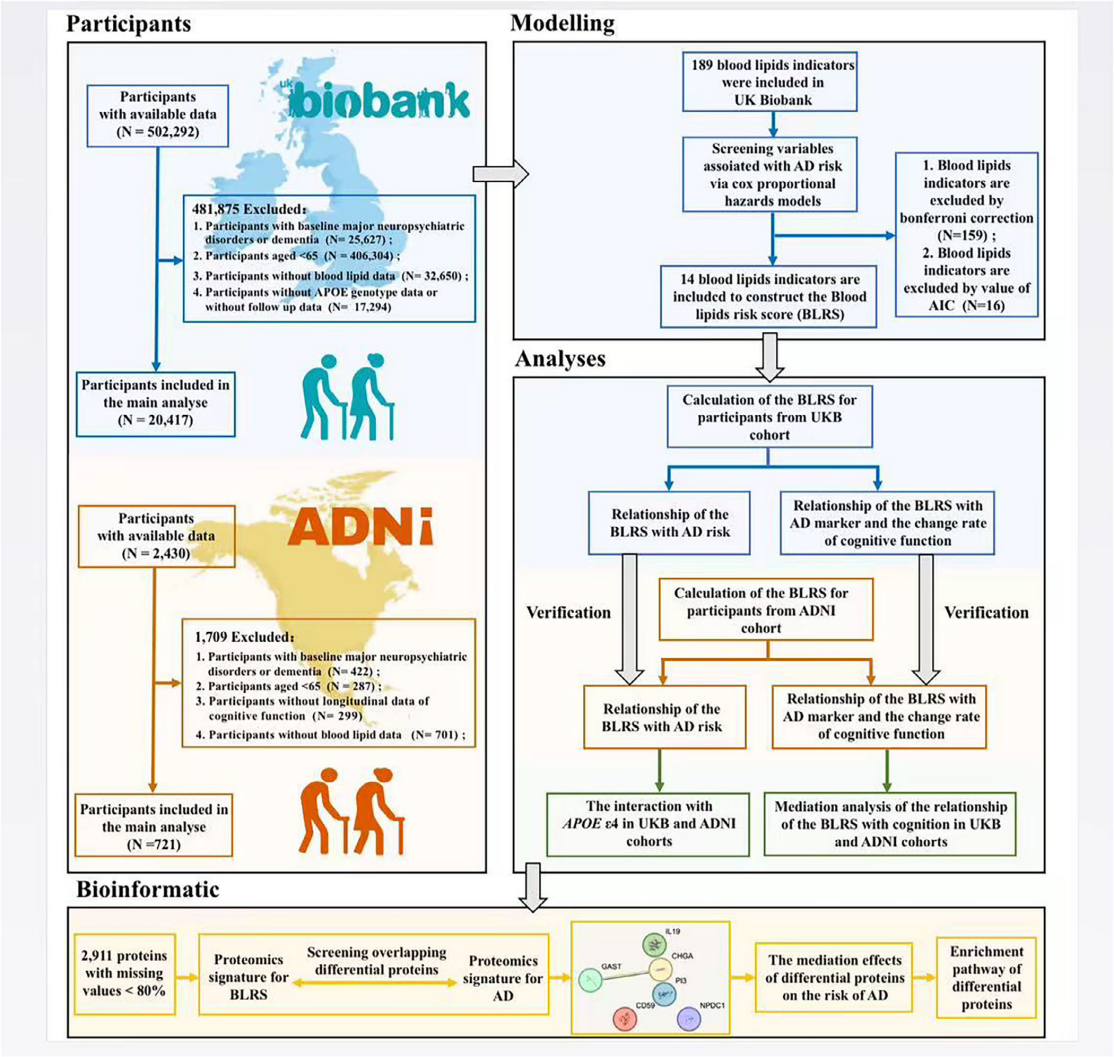# A blood lipidomic score and Alzheimer’s disease: associations and mediating mechanisms from two cohort studies

**DOI:** 10.1002/alz70861_108128

**Published:** 2025-12-23

**Authors:** Wei Xu, Chen‐Chen Tan, Liangyu Huang, Lan Tan

**Affiliations:** ^1^ Qingdao Municipal Hospital, Qingdao University, Qingdao, Shandong China; ^2^ Qingdao Municipal Hospital, Qingdao, 266071 China; ^3^ Qingdao Municipal hospital, Qingdao university, Qingdao, Shandong China

## Abstract

**Background:**

Dyslipidemia is a risk factor for Alzheimer's disease (AD). This study was to develop a blood lipid score (BLS) of lipidomics and investigate its associations with incident risk and endophenotypes of AD in older adults.

**Methods:**

The BLS associated with AD was developed using Cox proportional hazard regression. The construction cohort study enrolled 20,417 non‐demented adults who were aged ≥ 65 years and had blood lipidomic data from the UK Biobank. The BLS was replicated using data from the validation cohort Alzheimer's Disease Neuroimaging Initiative (ADNI, N = 721). Linear mixed‐effects models were used to test the associations of BLS with changing rates of AD cerebrospinal fluid (CSF) biomarkers, brain volumes, and cognitive function. The mediation effects and the additive and multiplicative interactions of BLS with *APOE* ε4 status were explored. Finally, causal mediation, proteomic, and bioinformatic analyses were conducted to investigate the underlying peripheral.

**Results:**

Of 251 lipidomic components, fourteen lipid components associated with AD risk were selected to construct BLS. In both the UKB (hazard ratio [HR] = 1.43, *p* < 0.001) and ADNI (HR = 2.22, *p* < 0.001), higher BLS was significantly associated with an increased risk of AD. Individuals with high BLS tended to have faster cognitive decline (Fluid Intelligence, *p* = 0.075 for UKB, ADAS‐13, *p* = 0.004 for ADNI) in the UKB and showed increasing rates of hippocampal atrophy (P = 0.005), entorhinal atrophy (P = 0.016), and CSF *p* ‐tau181 elevation (P = 0.033) in ADNI. Significant interaction of BLS × *APOE* ε4 was observed and the abovementioned associations were more pronounced in *APOE* ε4 carriers. Hippocampus volume and CSF tau proteins partially mediated the associations of BLS with cognitive decline (proportions ranging from 15.9% to 35.1%). Neuroendocrine‐related pathways, cytokines and immune responses, as well as the activation and regulation of the complement system could serve as potential mechanisms underlying the association between BLRS with AD.

**Conclusions:**

We developed a blood lipid score which could predict incident AD in two independent cohorts. The presence of the *APOE* ε4 exacerbated the association of dyslipidemia with AD risk. Neurodegeneration and immune system could be the mediating mechanisms.